# Cognitive Improvement and Microbiota–Gut–Brain Axis Regulation by *Lycium barbarum* Polysaccharides and Glycopeptide in Laboratory-Kenneled Poodles

**DOI:** 10.3390/microorganisms14040940

**Published:** 2026-04-21

**Authors:** Haoran Yan, Miaomiao Zhang, Chuchen Gui, Huiwen Huang, Wenhao Wu, Zhaokun Chen, Yuansheng Wu, Shaohao Chen, Hongcan Huang, Huixian Lin, Yan Guo, Baichuan Deng, Lingna Zhang

**Affiliations:** Laboratory of Companion Animal Science, Department of Animal Science, South China Agricultural University, Guangzhou 510642, China; 13680730396@163.com (H.Y.); zhangmiao7839@163.com (M.Z.); m18978024616@163.com (C.G.); 13138228231@163.com (H.H.); 19979815011@163.com (W.W.); 13640744747@163.com (Z.C.); chocolaty-cone@163.com (Y.W.); csh280101@163.com (S.C.); hongcan_huang@163.com (H.H.); 14754953648@163.com (H.L.); gy@scau.edu.cn (Y.G.)

**Keywords:** cognition, dog, *Lycium barbarum* glycopeptide, *Lycium barbarum* polysaccharides, gut microbiota, chronic stress

## Abstract

Kennel-housed dogs may experience chronic stress affecting cognition. This study compared the effects of *Lycium barbarum* polysaccharides (LBP) and glycopeptide (LbGP) on cognitive function in laboratory-kenneled poodles. Eighteen dogs were assigned to CON, LBP, or LbGP groups for 42 days. Cognitive tests were performed and serum, saliva, and feces were collected for subsequent analysis. Both supplements improved cognitive performance (e.g., increased the correct rate in the cylinder test by approximately 40.0%, both *p* < 0.001). LBP enriched beneficial bacteria (*Faecalibacterium* and *Bacteroides*, *p* < 0.05), reduced pathogens (*Romboutsia* and *Terrisporobacter*, *p* < 0.05), and predominantly influenced the indole pathway of tryptophan metabolism. LbGP specifically decreased *Escherichia-Shigella* and *Corynebacterium*, increased fecal SCFAs, and mainly targeted the 5-HT pathway. Both treatments regulated immune function (i.e., elevated IL-6 and IL-10) and antioxidant capacity; LBP significantly increased serum superoxide dismutase (SOD) levels by 6.8% (*p* < 0.01) and BDNF levels by 13.5% (*p* < 0.05), while LbGP elevated (*p* < 0.01) glutathione peroxidase (GSH-Px) levels by 20.9% and reduced salivary cortisol levels by 14.2% (*p* < 0.01). Overall, LBP and LbGP support canine cognition through distinct microbiota-related mechanisms, likely via the microbiota–gut–brain axis, suggesting their potential as functional feed additives for cognitive health.

## 1. Introduction

A survey study of 13,700 pet dogs in Finland indicated that approximately 32% of pet dogs experience anxiety [1]. Domestic dogs can encounter various challenges in daily life related to their environment and interactions with owners (e.g., unfamiliar environments, loud noises, limited social interaction, irregular schedules, and encounters with strangers) [2]. Although individual responses may vary, prolonged exposure to such conditions may be associated with behavioral and physiological changes in dogs, including the display of aggressive or depressive behaviors, cognitive alterations, and skin conditions [3,4]. Research indicates that as husbandry conditions become more restrictive, the associated physiological and behavioral changes in dogs become more pronounced, particularly for those maintained in standardized laboratory environments [5]. Current strategies to support canine behavioral health include medication, calming pheromones, and behavior modification. However, these approaches have limitations [6,7,8]. For instance, fluoxetine, a common anxiolytic drug, can induce adverse effects such as vomiting, diarrhea, and lethargy [9]. Similarly, the effect of appeasing pheromones is variable across individuals and can be inadequate in high-stress settings [10]. Behavior modification is time-consuming and labor-intensive, and often requires concurrent administration of psychotropic medications [11]. Therefore, more efficient and convenient treatment approaches are needed. The use of functional additives in nutritional interventions has garnered significant research interest and is being increasingly explored [12,13,14,15].

*Lycium barbarum* polysaccharides (LBP) are complex polysaccharide mixtures extracted from goji berries that are primarily composed of various monosaccharides (e.g., arabinose, galactose, glucose), forming highly branched sugar chains with complex glycosidic bond linkages [16]. The application of LBP in functional foods and pharmaceutical fields is common, particularly due to their antioxidant, anti-inflammatory, neuroprotective, anticancer, and immunomodulatory activities [17]. The LPS/TLR4/TRIF/NF-κB axis has been proposed to play key roles in the regulation of gut microbiota and intestinal barrier by polysaccharides [18]. A previous study demonstrated that LBP reversed chronic unpredictable stress (CUS) induced impairments on spatial learning and memory in rodents, as assessed by the Morris water maze test, potentially through restoring the hippocampal BDNF signaling pathway, alleviating morphological damage in the hippocampus, and reducing serum corticosterone levels [19]. *Lycium barbarum* glycopeptide (LbGP) is a glycoprotein further isolated and purified from LBP, the monosaccharide composition of which includes arabinose (Ara), galactose (Gal), and glucose (Glc) [20]. As the most promising monomeric component in goji fruit, LbGP has been extensively studied in the treatment of various conditions including cancer, recurrent chronic colitis, viral influenza, Parkinson’s disease, and anxiety disorders [21,22,23]. A previous study found that LbGP alleviated anxiety-like and depressive behaviors in mice subjected to chronic restraint stress [22]. This effect was potentially achieved by reducing oxidative stress and lipid peroxidation in the medial prefrontal cortex (mPFC), specifically through the inhibition of the local ferroptosis pathway [22]. These findings shed light on the possible mechanism by which LBP and LbGP influence cognitive function.

The gut microbiota influences brain function primarily through the microbiota–gut–brain (MGB) axis, a bidirectional communication system [24]. Zhang et al. (2025) recently reviewed microbiota-targeted strategies for neurocognitive regulation and demonstrated that gut-oriented interventions (e.g., probiotics, prebiotics, and dietary polysaccharides) modulate cognitive function via neural, endocrine, metabolic, and immune pathways of the MGB axis [25]. This system connects the gut and the central nervous system via multiple pathways [26]. For example, studies have shown that metabolites from specific gut microbiota, such as *Bifidobacterium dentium* (*B. dentium*) following intestinal colonization stimulated the production of serotonin from enterochromaffin cells (ECs), which subsequently acts on the local 5-HT receptors and the serotonin transporter (SERT) on adjacent intestinal cells and neurons, leading to the upregulation of hippocampal 5-HT receptor 2a expression in mice and the amelioration of their species-typical repetitive and anxiety-like behaviors [27]. Cheng et al. (2022) further demonstrated, using multi-compartment metabolomics, that specific gut bacterial families (e.g., Marinifilaceae and Akkermansiaceae) correlate with metabolite profiles in the feces, serum, and cortex of APP/PS1 mice, providing direct evidence for metabolite-mediated communication along the MGB axis [28].

Although results of previous studies have indicated the potential role of LBP in protecting the heart and liver of dogs, its effects and mechanisms on regulating anxiety and cognitive function in dogs remain to be explored [29,30]. As for LbGP, research on its applications in dogs is relatively limited. Despite the growing body of evidence supporting the cognitive benefits of LBP and LbGP in rodent models of stress and neurodegeneration, to the best of our knowledge, no study has systematically evaluated their effects on cognitive function in dogs, particularly under laboratory-kennel conditions. Furthermore, unlike polysaccharides, glycopeptides not only provide specific sugar moieties but also carry specific information regarding the amino acid sequence. Bioactive peptides have undergone natural selection, which enhances their in vivo stability [31]. Therefore, the bioactivity of LbGP has been shown to be stronger compared to LBP [32].

This study compared the effects of LBP and LbGP on stress and cognition of laboratory-kenneled dogs and explored the underlying mechanisms focusing on the regulation of gut microbiota and metabolism, as well as the immune function and antioxidant capacity. This comparison aims to provide theoretical support for future research and selective application.

## 2. Materials and Methods

### 2.1. Animals and Housing

The entire trial was conducted at Qingke Biotechnology Co., Ltd. (Guangzhou, China). The trial included 18 miniature poodles, around 6 years old, consisting of 9 males and 9 females, with an average initial BW of 6.51 ± 1.56 kg. These dogs had been housed at the facility for approximately 5 years prior to the study in Qingke Biotechnology Co., Ltd., and all dogs had been spayed or neutered. They were individually housed in standard laboratory cages within the same room. In addition to routine contact with caretakers, the dogs had daily access to outdoor environments (approximately one hour) and opportunities for social interaction with conspecifics. A chew toy (made of thermoplastic rubber) was provided in each cage. The dogs were housed in a standardized laboratory kennel (80 cm × 80 cm × 100 cm) environment with routine management protocols. Throughout the trial, a predetermined quantity of food was provided to the dogs at 09:00 and 16:00 each day, while drinking water was made available *ad libitum*. Cages and the room were cleaned daily and disinfected weekly. Trained personnel performed daily monitoring of food and water intake as well as behavioral performance, with body weight measured weekly. The criteria of the humane endpoint include body weight loss exceeding 20% of baseline, complete refusal of food and water for 24 consecutive hours, severe self-injurious behavior (e.g., persistent self-mutilation), and presentation of severe neurological signs such as inability to stand, convulsions [33]. The animal meeting any of the above criteria should be immediately evaluated and treated by a veterinarian, and euthanized when determined by the veterinarian to be absolutely necessary. None of the dogs in the current study reached humane endpoint during the experimental period.

### 2.2. Treatment Administrations and Sample Collection

The basal diet was produced by Qingke Biotechnology Co., Ltd. (Guangzhou, China) and complied with the nutritional recommendations for dogs established by the Association of American Feed Control Officials (AAFCO, 2017). The ingredient composition and chemical analysis are presented in Table S1. Dogs were allocated into three treatment groups: a basal diet group (Control, CON, n = 6, BW = 6.30 ± 1.47 kg), a group with *Lycium barbarum* polysaccharides (LBP, n = 6, BW = 6.35 ± 1.46 kg), and a group fed with basal diet supplemented with *Lycium barbarum* glycopeptide (LbGP, n = 6, BW = 6.88 ± 1.93 kg). The dogs in the three groups were balanced for initial body weight and sex (3 males and 3 females per group). To minimize order and spatial effects, housing cage allocation, daily treatment administration, and the sequence of behavioral tests and biosample (saliva and blood) collection were all randomized and counterbalanced throughout the study. Prior to the commencement of the trial, all dogs had received necessary vaccinations and deworming treatments. Furthermore, they had not been administered any medication that could potentially affect the trial outcomes for at least one month prior to the start of the study.

**Figure 1:**
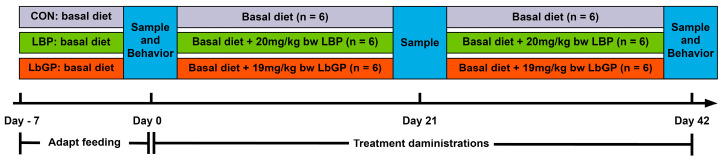
Timeline illustrating the experimental design, sampling time points, and behavioral test schedule. CON: control group (basal diet); LBP: basal diet supplemented with 20 mg/kg BW *Lycium barbarum* polysaccharides; LbGP: basal diet supplemented with 19 mg/kg BW *Lycium barbarum* glycopeptide.

Following a 7-day adaptation, CON group dogs received the basal diet plus empty size 1 starch capsules daily, whereas the LBP and LbGP groups were fed the basal diet supplemented with size 1 starch capsules containing 20 mg/kg BW of LBP (90% purity, Shaanxi Zhenghe Pharmaceutical Bioengineering Co., Ltd., Shaanxi, China) and 19 mg/kg BW of LbGP (95% purity, Ningxia Tianren Goji Biotechnology, Ningxia, China), respectively [34,35]. These dosages were based on previous studies in mice and converted according to the Meeh-Rubner equation, taking into account the body surface area of both mice and dogs. Prior to the daily afternoon feeding, capsules (either placebo or containing the compound) were administered to the dogs. The sampling time points and behavioral test timeline for this trial were shown in Figure 1.

After an 8-h fast, 5 mL of blood was collected from the forelimb vein of each dog into blood collection tubes on Day 0, Day 21, and Day 42. The tubes were tilted and allowed to stand for 30 min, then centrifuged at 1500× *g* for 15 min at room temperature. The supernatant was aliquoted into microcentrifuge tubes and stored at −80 °C for further analysis. On Day 0 and Day 42, saliva was collected from fasted dogs before blood collection between 8:00 a.m. and 10:00 a.m. Food and water were withheld for 1 h prior to saliva collection. The saliva samples were centrifuged at 1000× *g* for 5 min, and the supernatant was carefully collected, protected from light, and rapidly transferred to a −80 °C for further analysis. On Day 0, Day 21, and Day 42, fresh feces excreted within 10 min were collected. Using a fecal collector, uncontaminated samples from the interior portion of the feces were obtained. The samples were aliquoted into cryovials and stored at −80 °C for further analysis.

### 2.3. Behavior and Sample Analyses

#### 2.3.1. Assessment of Cognitive Ability

On both the start (Day 0) and end (Day 42) of the trial, the dogs underwent behavioral tests to evaluate their problem-solving skills, social cognition, inhibitory control, and memory. Due to a lack of interest in food rewards among some of the trial dogs, only 5 dogs from each group participated in the cognitive testing (the excluded dogs were all females). All dogs exhibited normal vision, hearing, and motor function. Testing methods referred to the study proposed by Saara Junttila et al. (2022), with minor adaptations made to accommodate the size of the trial dogs [36]. Specifically, the dogs underwent six tasks in a randomized order within a spacious and quiet area measuring 5.20 m × 4.80 m. All tests used freeze-dried duck breast as food rewards. The tasks included the cylinder test, human gesture test, V-detour test, logical reasoning test, memory test, and memory vs. gesture test. The experimental area was thoroughly scrubbed with disinfectant daily, both one day prior to and throughout the trial period. Between tests involving different dogs, the arena and associated apparatus were sprayed with 75% alcohol to eliminate residual odors from the previous animal. The area was then ventilated for 5 min to allow the scent to dissipate and wiped before the next dog was introduced.

**Figure 2:**
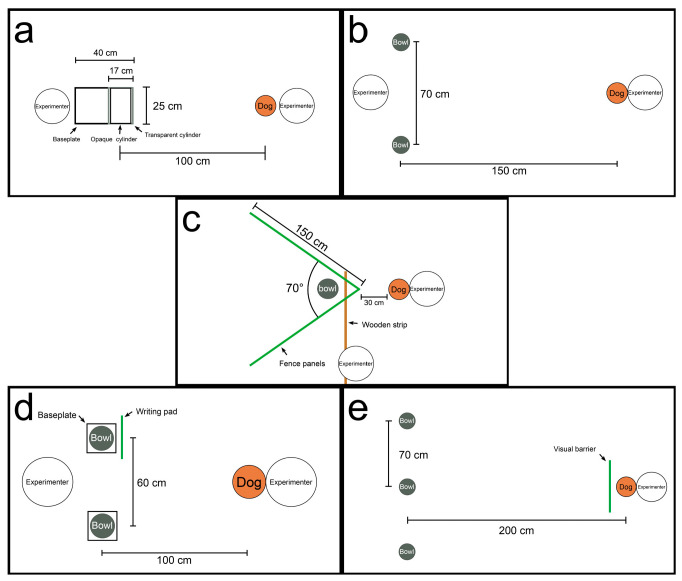
Cognitive Ability Test. (**a**) Cylinder test; (**b**) Human gestures and Memory vs. gesture; (**c**) V-detour; (**d**) Logical reasoning; (**e**) Memory.

The cylinders in the cylinder test were customized according to the size of the dogs (Figure 2a). All components were made of acrylic, including a transparent cylinder (inner diameter of 16.4 cm, thickness of 0.6 cm, length of 25 cm), an opaque white cylinder (inner diameter of 15.4 cm, thickness of 0.6cm, and length of 25 cm), and a baseplate measuring 25 × 40 cm with a thickness of 0.3 cm. The transparent cylinder was affixed to the baseplate using adhesive tape, while the opaque cylinder was inserted inside the transparent one. During the test, the dog was positioned approximately 1 m away from the cylinder. The remaining procedures followed the original protocol. The average percentage of correct responses per group was used to measure motor inhibitory response. In both the human gesture test and the memory vs. gesture test, the dog started facing the experimenter from a distance of 1.5 m. Two bowls were placed approximately 70 cm apart on the floor in front of the experimenter (Figure 2b). The remaining procedures followed the original protocol. Social cognitive ability was assessed using the average percentage of correct responses per group in the human gesture test. For the memory vs. gesture test, the dogs’ choice preferences were recorded. In the V-detour test, the V-shaped barrier was constructed using two pet fence panels, each 150 cm long and 50 cm high, arranged at a 70° angle and secured with a 120 cm long wooden strip at the vertex (Figure 2c). The dog started the test from a point about 30 cm from the apex of the V-barrier. The remaining procedures followed the original protocol. The average time taken to obtain the food reward per group was used to evaluate spatial problem-solving ability and inhibitory control. The test procedure of logical reasoning fully followed the original protocol. The average percentage of correct responses per group was used to measure logical reasoning ability (Figure 2d). In the memory test, three identical opaque bowls were placed facing upward in a straight line on the floor, spaced approximately 70 cm apart (Figure 2e). The dog began the test from a position 2 m away from the central bowl. The remaining procedures followed the original protocol. The average percentage of correct responses per group was used to assess short-term memory capacity.

#### 2.3.2. Serum Biochemistry Analyses

Serum biochemical parameters were measured using a fully automated multifunctional biochemistry analyzer (Seamaty-120VP, Seamaty Technology Co., Ltd. Chengdu, China) and corresponding assay kits (Jiangsu Aidisheng Biotechnology Co., Ltd., Jiangsu, China) in accordance with the manufacturer’s instructions. Levels of tumor necrosis factor-alpha (TNF-α), interleukin-1 beta (IL-1β), IL-6, IL-10, interferon-gamma (IFN-γ), immunoglobulin A (IgA), superoxide dismutase (SOD), glutathione peroxidase (GSH-Px), catalase (CAT), brain-derived neurotrophic factor (BDNF), neurofilament light chain (NfL), as well as cortisol in saliva, were measured using commercial canine-specific enzyme-linked immunosorbent assay (ELISA) kits (Jiangsu Meimian Industrial Co., Ltd., Jiangsu, China). All procedures were performed in accordance with the manufacturers’ protocols. Absorbance was read using a multifunctional microplate reader (Thermo Fisher Scientific, Waltham, MA, USA) to determine the concentrations of the respective biomarkers.

#### 2.3.3. Fecal Short-Chain Fatty Acids (SCFAs) and Branched-Chain Fatty Acids (BCFAs) Analysis

After fully thawed while placed on ice, approximately 0.2 g of each sample was weighed into a 2 mL microcentrifuge tube, followed by the addition of 1 mL of ultrapure water obtained from a Flex3 ultrapure water purification system (ELGA LabWater, High Wycombe, UK). The mixture was vortexed for 5 min to ensure homogeneity. Subsequently, it was subjected to ultrasonic disruption for 10 min in an ice bath and then centrifuged at 18,000× *g* and 4 °C for 10 min. The supernatant was carefully collected into a new microcentrifuge tube. A volume of 20 µL of 25% metaphosphoric acid (Tianjin Kermel Chemical Reagent Co., Ltd., Tianjin, China) and 0.25 g of anhydrous sodium sulfate (Shanghai Enn Chemical Technology Co., Ltd., Shanghai, China) were added to the supernatant. The mixture was vortexed for 2 min for acidification and salt-out extraction. Under a fume hood, 1 mL of methyl tert-butyl ether (MTBE, Shanghai Aladdin Biochemical Technology Co., Ltd., Shanghai, China) was added to each sample, followed by vortexing for 5 min. The samples were then centrifuged at 18,000× *g* and 4 °C for 5 min. The upper MTBE extraction layer was collected using a 1 mL disposable syringe (Taizhou Ming’an Medical Instrument Co., Ltd., Taizhou, China), filtered through a 0.22 µm micropore membrane (Millipore, Billerica, MA, USA), and transferred into a vial with an insert for subsequent analysis. Quantification of SCFAs and BCFAs was performed using gas chromatography–mass spectrometry (GC–MS; GC-MS-QP2020 system, Shimadzu, Tokyo, Japan) according to the method previously used in our laboratory [37].

#### 2.3.4. Feces Microbiota Analysis

16S rRNA gene sequencing and gut microbiota analysis were performed according to the method described by Bai et al. (2026) [38], with minor modifications. Briefly, total genomic DNA was extracted from fecal samples using the E.Z.N.A.^®^ Soil DNA Kit (Omega Bio-tek, Norcross, GA, USA). The V3–V4 region of the 16S rRNA gene was amplified with primers 338F and 806R. PCR products were purified, quantified, and sequenced on an Illumina NextSeq 2000 platform. Raw reads were quality-filtered, merged, and clustered into OTUs at 97% similarity. Taxonomic assignment was performed using the RDP Classifier against the Silva database. All bioinformatic analyses were carried out on the Majorbio Cloud platform. For each OTU, the most abundant sequence was selected as the representative. To account for differences in sequencing depth across samples, the read counts were rarefied to 20,000 per sample, resulting in an average Good’s coverage of 99.09%. Taxonomic assignment of the representative OTU sequences was performed using the RDP Classifier (version 2.2) against the Silva v138 16S rRNA gene database with a confidence threshold of 0.7 [39].

Bioinformatic analysis of the feces microbiota was carried out using the Majorbio Cloud platform (https://cloud.majorbio.com) [40]. Alpha diversity indices (observed OTUs, Chao1 richness, Shannon, and Simpson) were computed using Mothur v1.30.1 [41]. Group comparisons of alpha diversity were performed with the Kruskal–Wallis rank-sum test, followed by false discovery rate (FDR) correction for multiple comparisons and Dunn’s post-hoc test. Beta diversity was assessed via principal coordinate analysis (PCoA) based on weighted UniFrac distances using the Vegan v2.5-3 package, and the analysis of similarities (ANOSIM) was applied to evaluate the proportion of variation explained by treatment and its statistical significance. Differential bacterial abundances at the phylum, family, and genus levels between each treatment group and the CON group were analyzed using the Wilcoxon rank-sum test, with CON as the reference. *p*-values were adjusted for multiple comparisons using Holm’s method. All statistical analyses were performed with R (version 3.3.1, R Core Team, Vienna, Austria; stats package) and Python (version 1.0.0, Python Software Foundation, Wilmington, DE, USA; scipy package). Linear discriminant analysis (LDA) effect size (LEfSe) (http://huttenhower.sph.harvard.edu/LEfSe) was used to identify significantly enriched bacterial taxa from phylum to genus (LDA score > 2, *p* < 0.05) among the groups [42].

#### 2.3.5. Targeted Metabolome Analyses of Serum and Feces

Targeted metabolomics of serum and feces was performed according to the method previously used in our laboratory [15], with minor modifications. Briefly, 200 μL of sample (serum or fecal supernatant) was mixed with 800 μL of methanol, vortexed, and centrifuged at 23,500× *g* for 15 min at 4 °C. The supernatant was evaporated under nitrogen, reconstituted in 200 μL methanol, ultrasonicated, and centrifuged again under the same conditions. The resulting supernatant was filtered (0.22 μm) for UPLC-Orbitrap-MS/MS analysis [43]. Raw data preprocessing and metabolite identification were performed using Compound Discoverer 2.1 (Thermo Fisher Scientific, Waltham, MA, USA) by searching the mzCloud and mzVault libraries.

Metabolites involved in the Trp pathway (Table S2) were quantified by targeted metabolomics. Stock standard solutions were prepared by dissolving reference compounds in methanol-water (1:1) and serially diluted to concentrations of 1000, 500, 100, 10, 1, 0.5, 0.1, 0.01, and 0.001 ng/mL. These standards were used to generate calibration curves via UPLC-Orbitrap-MS/MS according to the method in [44]. The resulting raw data were then processed with Xcalibur software (version 4.3, Thermo Fisher Scientific, Waltham, MA, USA) to calculate metabolite concentrations based on the corresponding standard curves.

#### 2.3.6. Statistical Analysis

All statistical analyses were performed with SPSS software v26.0 (IBM Corporation, Chicago, IL, USA). One-way analysis of variance (ANOVA) was used to compare inter-group differences at the same experimental time point, with LSD employed for post-hoc tests. For continuous variables that did not conform to a normal distribution, the Kruskal–Wallis H test was used to compare inter-group differences at the same experimental time point. For data satisfying normality and homogeneity of variance, a two-way repeated measures analysis of variance (ANOVA) followed by LSD post hoc tests was applied to evaluate both between-group differences at each time point and within-group changes across different time points. For variables that did not meet normality assumptions, a generalized linear mixed model (GLMM) was used to assess between-group differences at consistent time points and within-group variations over time. The model included time points (i.e., time), groups (i.e., treatment administrations, TRT), and the interaction between the two factors (time × TRT) as fixed effects, random by dog.

A *p*-value < 0.05 was considered statistically significant, and a *p*-value between 0.05 and 0.10 was regarded as indicating a statistical trend. Unless otherwise specified, the data are presented as mean ± standard error of the mean (SEM). All figures were prepared using GraphPad Prism v10.0 (GraphPad Software Inc., San Diego, CA, USA) and Adobe Illustrator 2022 (Adobe, San Jose, CA, USA).

## 3. Results

### 3.1. Cognitive Ability

**Figure 3:**
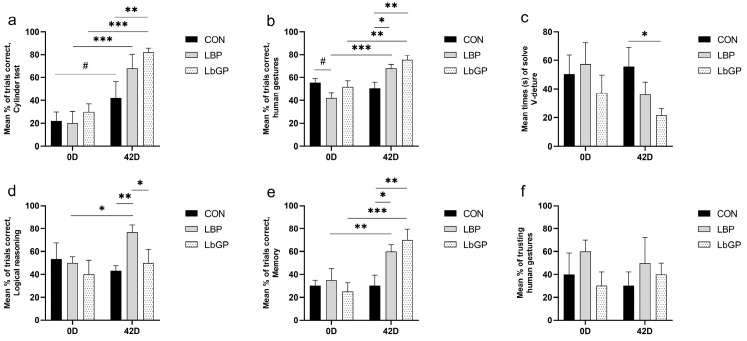
Effects of LBP and LbGP on cognitive performance in dogs. (**a**) Cylinder test; (**b**) Human gestures; (**c**) V-detour; (**d**) Logical reasoning; (**e**) Memory; (**f**) Memory vs. gesture. 0 D refers to the start of the experiment (Day 0), and 42 D indicates the end of the experimental period (Day 42). CON: control group (basal diet); LBP: basal diet supplemented with 20 mg/kg BW *Lycium barbarum* polysaccharides; LbGP: basal diet supplemented with 19 mg/kg BW *Lycium barbarum* glycopeptide (n = 5 per group for cognitive testing, initial n = 6). Data are presented as mean ± SEM. Asterisks (*) indicate statistically significant differences compared to baseline or control (* *p* < 0.05, ** *p* < 0.01, *** *p* < 0.001), and the hash symbol (#) denotes a statistical trend (# *p* < 0.10).

As shown in Figure 3a, a main effect of time was observed (*p* < 0.001), with a trend for time × TRT interaction for the cylinder test. A significant time × TRT interaction was found in the human gestures test (*p* < 0.01; Figure 3b), while the V-detour test showed a main effect of TRT (*p* < 0.05; Figure 3c). The logical reasoning test revealed a time × TRT interaction (*p* < 0.05; Figure 3d), whereas a highly significant interaction of time × TRT was detected in the memory test (*p* < 0.01; Figure 3e). No significant effects of LBP or LbGP were observed on choice propensity in the memory vs. gesture test (Figure 3f).

Specifically, both LBP and LbGP supplementation improved the performance of poodles from Day 0 to Day 42 in the cylinder test (+48.0%, *p* < 0.001; +52.0%, *p* < 0.001) and the human gestures test (+24.0%, *p* < 0.001; +23.3%, *p* < 0.01). In the V-detour test, the LbGP group had shorter completion times than the CON group at Day 42 (−61.3%, *p* < 0.05). For logical reasoning, LBP resulted in higher accuracy from Day 0 to Day 42 (+26.7%, *p* < 0.05). Both supplements enhanced memory test performance by Day 42 (+25.0%, *p* < 0.01; +45.0%, *p* < 0.001).

### 3.2. Fecal SCFAs and BCFAs

**Figure 4:**
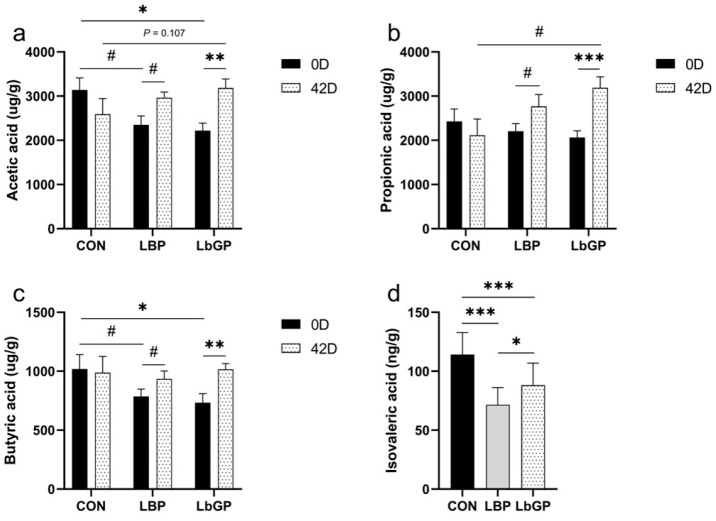
Effects of LBP and LbGP on fecal SCFAs and BCFAs concentrations in dogs. (**a**) Acetic acid; (**b**) Propionic acid; (**c**) Butyric acid; (**d**) Isovaleric acid. 0 D refers to the start of the experiment (Day 0), and 42 D indicates the end of the experimental period (Day 42). CON: control group (basal diet); LBP: basal diet supplemented with 20 mg/kg BW *Lycium barbarum* polysaccharides; LbGP: basal diet supplemented with 19 mg/kg BW *Lycium barbarum* glycopeptide. Data are presented as mean ± SEM. Asterisks (*) indicate statistically significant differences compared to baseline or control (* *p* < 0.05, ** *p* < 0.01, *** *p* < 0.001), and the hash symbol (^#^) denotes a statistical trend (^#^ *p* < 0.10).

As shown in Figure 4, a trend toward a main effect of time on acetic acid levels was observed (*p* < 0.1), and the time × TRT interaction effect was significant (*p* < 0.05). For propionic acid and butyric acid, the main effect of time was significant (*p* < 0.05). The time × TRT interaction effect was observed for propionic acid (*p* < 0.01) and butyric acid (*p* < 0.05). The main effect of TRT on isovaleric acid was observed (*p* < 0.001, Figure 4d).

Regarding fecal acetic acid (Figure 4a), as the trial progressed, the LBP group showed a trend toward increased acetic acid levels (*p* < 0.1); and the LbGP group showed an increase in acetic acid levels (*p* < 0.01). For fecal propionic acid (Figure 4b), the LBP group showed a trend toward increased levels (*p* < 0.1), while the LbGP group showed an increase (*p* < 0.001). For fecal butyric acid (Figure 4c), the LBP group showed a trend toward increased levels (*p* < 0.1), whereas the LbGP group showed an increase (*p* < 0.01).

### 3.3. Structure and Composition of the Fecal Microbiota

**Figure 5:**
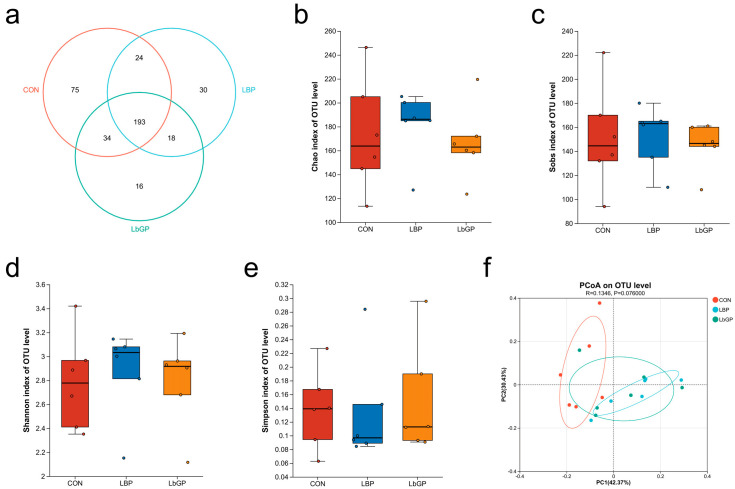
Effects of LBP and LbGP on species composition, alpha-diversity, and beta-diversity of the fecal microbiota in dogs. (**a**) Venn diagram illustrating shared and unique operational taxonomic units (OTUs) among groups; (**b**) Chao index; (**c**) Sobs index; (**d**) Shannon index; (**e**) Simpson index; (**f**) Principal coordinates analysis (PCoA). CON: control group (basal diet); LBP: basal diet supplemented with 20 mg/kg BW *Lycium barbarum* polysaccharides; LbGP: basal diet supplemented with 19 mg/kg BW *Lycium barbarum* glycopeptide. Data are presented as mean ± SD.

Analysis of the fecal microbiota revealed both shared and treatment-specific responses. Venn diagram analysis indicated that 193 OTUs were common to all three groups, while the CON, LBP, and LbGP groups contained 75, 30, and 16 unique OTUs, respectively (Figure 5a). No significant differences in alpha diversity, as assessed by the Chao, Sobs, Shannon, and Simpson indices were detected among the groups (all *p* > 0.10; Figure 5b–e). PCoA of beta diversity showed a trend toward structural separation (R = 0.135, *p* = 0.076; Figure 5f), with clear visual distinction between the CON and LBP groups and modest separation between the CON and LbGP groups.

**Figure 6:**
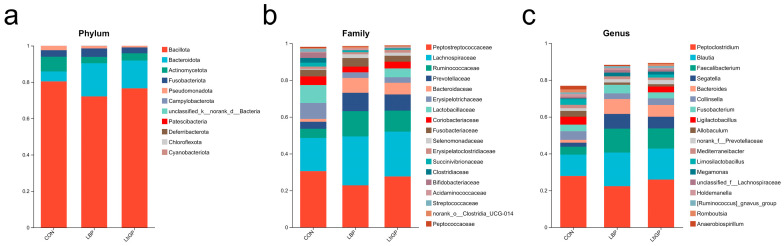
Fecal microbiota composition among CON, LBP, and LbGP groups. (**a**) Taxonomic profile at the phylum level; (**b**) Taxonomic profile at the family level; (**c**) Taxonomic profile at the genus level. CON: control group (basal diet); LBP: basal diet supplemented with 20 mg/kg BW *Lycium barbarum* polysaccharides; LbGP: basal diet supplemented with 19 mg/kg BW *Lycium barbarum* glycopeptide.

Taxonomic profiling demonstrated modulated microbial composition under different treatments (Figure 6). At the phylum level, all groups were predominantly composed of Bacillota, Bacteroidota, Actinomycetota, Fusobacteriota, and Pseudomonadota, with slight variations in relative abundance across groups (Figure 6a). Family-level analysis revealed high abundances of Peptostreptococcaceae, Lachnospiraceae and Ruminococcaceae in all groups, with the LBP and LbGP groups showing increased representation of Prevotellaceae and Bacteroidaceae (Figure 6b). At the genus level, *Peptoclostridium* and *Blautia* were highly abundant across groups. The LBP group exhibited increased relative abundance of *Faecalibacterium* and *Bacteroides*, while the LbGP group showed elevated *Segatella* and *Bacteroides* (Figure 6c).

**Figure 7:**
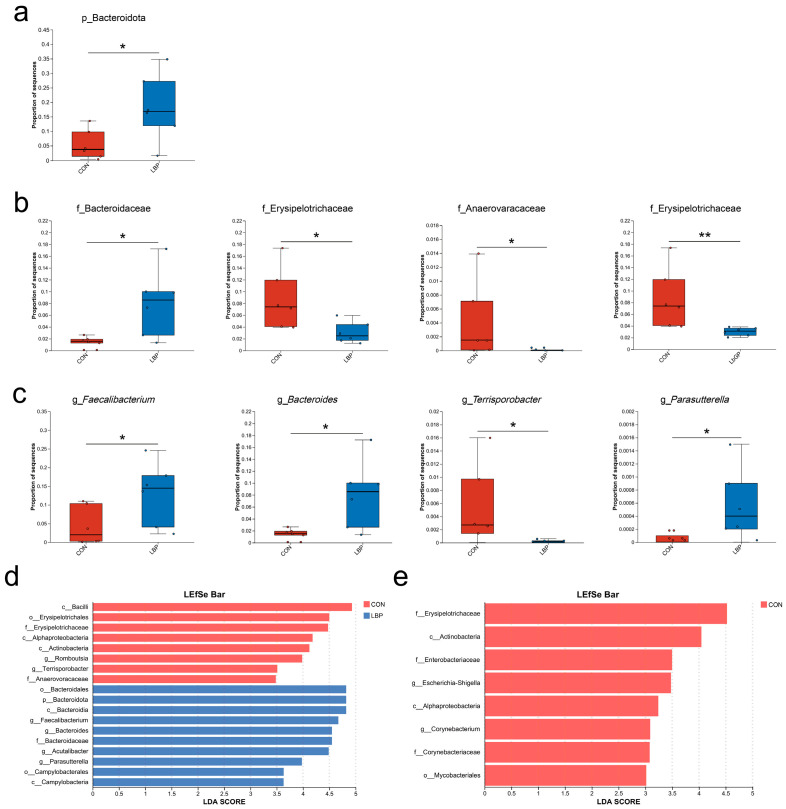
Differential gut microbial analysis between CON, LBP, and LbGP groups. (**a**) Differentially abundant microbes at the phylum level; (**b**) Differentially abundant microbes at the family level; (**c**) Differentially abundant microbes at the genus level; (**d**) Linear discriminant analysis (LEfSe) between CON and LBP groups; (**e**) Linear discriminant analysis (LEfSe) between CON and LbGP groups. CON: control group (basal diet); LBP: basal diet supplemented with 20 mg/kg BW *Lycium barbarum* polysaccharides; LbGP: basal diet supplemented with 19 mg/kg BW *Lycium barbarum* glycopeptide. Data are presented as mean ± SD. Asterisks (*) indicate statistically significant differences compared to baseline or control (* *p* < 0.05, ** *p* < 0.01).

Differential abundance analysis further highlighted treatment effects (Figure 7). The LBP group showed a significant increase in Bacteroidota at the phylum level compared to the CON group (*p* < 0.05; Figure 7a). At the family level, Bacteroidaceae was enriched in the LBP group, while Erysipelotrichaceae and Anaerovoracaceae were more abundant in the CON group (all *p* < 0.05). The CON group also showed markedly higher Erysipelotrichaceae abundance than the LbGP group (*p* < 0.01; Figure 7b). Genus-level analysis indicated higher abundances of *Faecalibacterium*, *Bacteroides*, and *Parasutterella* in the LBP group relative to the CON group (*p* < 0.05), whereas *Terrisporobacter* was enriched in the CON group (*p* < 0.05; Figure 7c).

LEfSe analysis confirmed these trends, indicating enrichment of *Faecalibacterium*, *Bacteroides*, *Acutalibacter*, and *Parasutterella* in the LBP group, and *Romboutsia* and *Terrisporobacter* in the CON group (Figure 7d). When compared to LbGP group, *Escherichia-Shigella* and *Corynebacterium* were significantly enriched in the CON group (Figure 7e).

### 3.4. Serum Biochemical Parameters

Serum biochemical parameters for the three groups are presented in Table 1. At both Day 0 and Day 42, all mean values fell within the normal reference ranges. No significant treatment effects were detected for any of the parameters. Compared to Day 0, supplementation with LBP reduced serum levels of calcium (Ca, *p* < 0.05), aspartate aminotransferase (AST, *p* < 0.05), the AST/ALT ratio (*p* < 0.05), albumin (ALB, *p* < 0.01), and total protein (TP, *p* < 0.01). In addition, LbGP supplementation reduced serum albumin (ALB, *p* < 0.05) and calcium (Ca, *p* < 0.05). A trend of increment in the AST/ALT ratio was observed in the CON group.

### 3.5. Serum Cytokines

**Figure 8:**
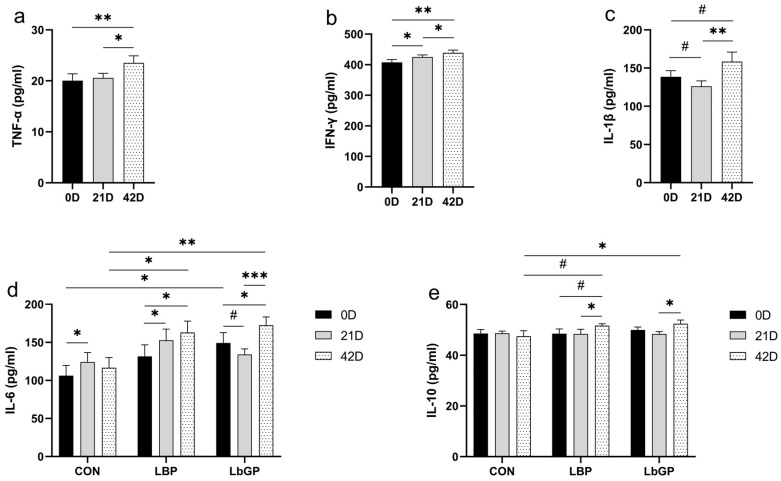
Effects of LBP and LbGP on serum cytokine levels in dogs. (**a**) Tumor necrosis factor-alpha (TNF-α); (**b**) Interferon-gamma (IFN-γ); (**c**) Interleukin-1 beta (IL-1β); (**d**) Interleukin-6 (IL-6); (**e**) Interleukin-10 (IL-10). 0 D refers to the start of the experiment (Day 0), and 42 D indicates the end of the experimental period (Day 42). CON: control group (basal diet); LBP: basal diet supplemented with 20 mg/kg BW *Lycium barbarum* polysaccharides; LbGP: basal diet supplemented with 19 mg/kg BW *Lycium barbarum* glycopeptide. Data are presented as mean ± SEM. Asterisks (*) indicate statistically significant differences compared to baseline or control (* *p* < 0.05, ** *p* < 0.01, *** *p* < 0.001), and the hash symbol (^#^) denotes a statistical trend (^#^ *p* < 0.10).

Serum levels of TNF-α and IFN-γ increased over time (*p* < 0.01; Figure 8a,b), while IL-1β showed only an increasing trend (*p* < 0.1; Figure 8c). A significant time × TRT interaction was observed for IL-6 (*p* < 0.05; Figure 8d). At baseline, IL-6 was higher in the LbGP group than in the CON group (*p* < 0.05). Post-trial, IL-6 levels increased in both the LBP and LbGP groups (both *p* < 0.05). At this endpoint, the LBP group had higher IL-6 than the CON group (*p* < 0.05), and the LbGP group exhibited even greater elevation (*p* < 0.01). For IL-10, a trend of time × TRT interaction was observed (*p* < 0.1; Figure 8e). The LBP group showed an increasing trend from baseline (*p* < 0.1) and tended to be higher than CON group at Day 42 (*p* < 0.1). In contrast, the LbGP group had higher IL-10 levels than the CON group (*p* < 0.05).

### 3.6. Serum Antioxidant Measures

**Figure 9:**
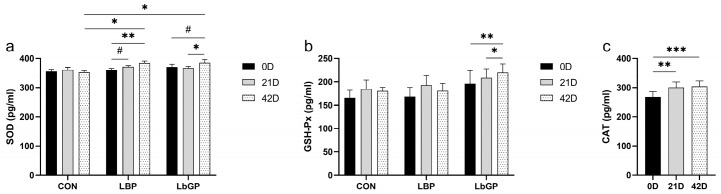
Effects of LBP and LbGP on serum antioxidant measures in dogs. (**a**) Superoxide dismutase (SOD); (**b**) Glutathione peroxidase (GSH-Px); (**c**) Catalase (CAT). 0 D refers to the start of the experiment (Day 0), and 42 D indicates the end of the experimental period (Day 42). CON: control group (basal diet); LBP: basal diet supplemented with 20 mg/kg BW *Lycium barbarum* polysaccharides; LbGP: basal diet supplemented with 19 mg/kg BW *Lycium barbarum* glycopeptide. Data are presented as mean ± SEM. Asterisks (*) indicate statistically significant differences compared to baseline or control (* *p* < 0.05, ** *p* < 0.01, *** *p* < 0.001), and the hash symbol (^#^) denotes a statistical trend (^#^ *p* < 0.10).

For serum SOD (Figure 9a), a significant time × TRT interaction was observed (*p* < 0.05). For serum GSH-Px and CAT (Figure 9b,c), only a significant main effect of time was detected (GSH-Px: *p* < 0.05; CAT: *p* < 0.001). Specifically, SOD in the LBP group showed a highly increase from baseline (*p* < 0.01), while a trend toward elevation was observed in the LbGP group (*p* < 0.1). After the trial, both supplemented groups exhibited higher SOD levels than the CON group (both *p* < 0.05). Although the time × TRT interaction was not significant (*p* > 0.10), increase in GSH-Px activity was detected in the LbGP group after the intervention (*p* < 0.01; Figure 9b).

### 3.7. Serum BDNF, NfL, Salivary and Serum Cortisol

**Figure 10:**
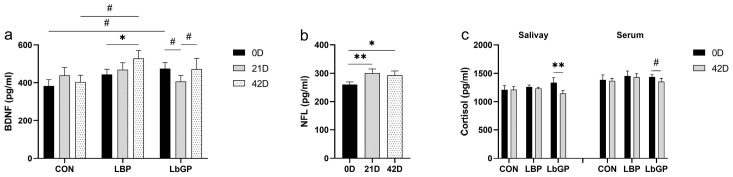
Effects of LBP and LbGP on serum BDNF, NfL, salivary and serum cortisol levels in dogs. (**a**) Brain-derived neurotrophic factor (BDNF); (**b**) Neurofilament light chain (NfL); (**c**) Salivary and serum cortisol. 0 D refers to the start of the experiment (Day 0), and 42 D indicates the end of the experimental period (Day 42). CON: control group (basal diet); LBP: basal diet supplemented with 20 mg/kg BW *Lycium barbarum* polysaccharides; LbGP: basal diet supplemented with 19 mg/kg BW *Lycium barbarum* glycopeptide. Data are presented as mean ± SEM. Asterisks (*) indicate statistically significant differences compared to baseline or control (* *p* < 0.05, ** *p* < 0.01), and the hash symbol (^#^) denotes a statistical trend (^#^ *p* < 0.10).

A time × TRT interaction was observed for serum BDNF (*p* < 0.1; Figure 10a). After the trial, BDNF increased compared to baseline in the LBP group (*p* < 0.05), but was unchanged in the LbGP group. Serum NfL levels increased over time (*p* < 0.05), with no effect of TRT or interaction (Figure 10b). A significant time × TRT interaction was found for salivary cortisol concentrations, with a decrease in the LbGP group post-treatment (*p* < 0.01; Figure 10c). Serum cortisol was unaffected by treatment, even though the LbGP group showed a trend of decrease (*p* < 0.1).

### 3.8. Targeted Metabolome Analyses

**Figure 11:**
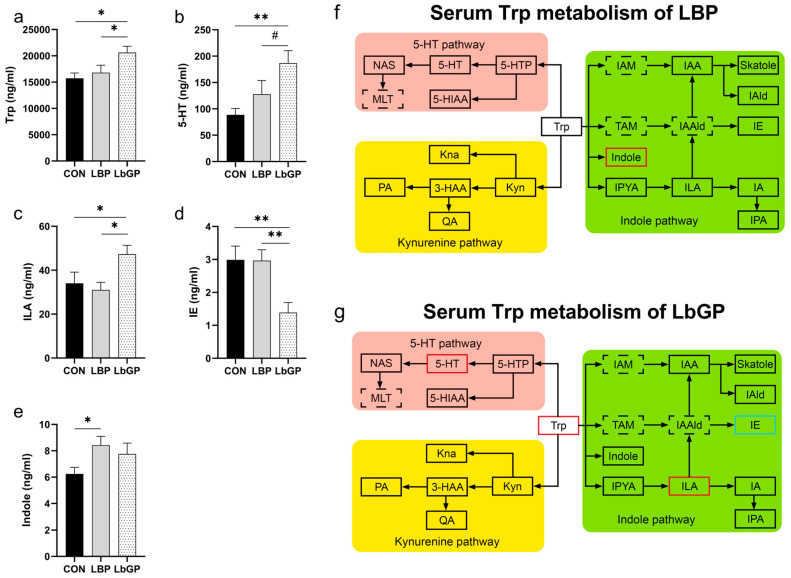
Effects of LBP and LbGP on Serum Metabolites of the Trp Metabolic Pathway on 42 D. (**a**) Trp, tryptophan; (**b**) 5-HT, 5-hydroxytryptamine; (**c**) ILA, indole-3-lactic acid. (**d**) IE, indole ethanol; (**e**) Indole, indole; (**f**) Serum Trp metabolism of LBP; (**g**) Serum Trp metabolism of LbGP. Black box: no statistical difference; red box: increase; blue box: decrease; dotted box: less than three samples were detected. CON: control group (basal diet); LBP: basal diet supplemented with 20 mg/kg BW *Lycium barbarum* polysaccharides; LbGP: basal diet supplemented with 19 mg/kg BW *Lycium barbarum* glycopeptide. Data are presented as mean ± SEM. Asterisks (*) indicate statistically significant differences compared to baseline or control (* *p* < 0.05, ** *p* < 0.01), and the hash symbol (^#^) denotes a statistical trend (^#^ *p* < 0.10). For a complete list of abbreviations of all detected Trp-related metabolites, see Supplementary Materials Table S2.

As shown in Figure 11, analysis of specific metabolites in the Trp metabolic pathway in dog serum on 42 D indicated that both LBP and LbGP exerted significant regulatory effects on particular metabolites within this pathway. Specifically, in the LbGP group, serum Trp levels were higher than those in the CON group (*p* < 0.05, Figure 11a). Concentrations of 5-HT was higher than those in the CON group (*p* < 0.01, Figure 11b), and showed a trend toward being higher than those in the LBP group (*p* < 0.10). Indole-3-lactic acid was higher than those in both the CON and LBP groups (both *p* < 0.05, Figure 11c). Indole ethanol levels were lower than those in both the CON and LBP groups (both *p* < 0.01, Figure 11d). In the LBP group, serum indole levels were higher than those in the CON group (*p* < 0.05, Figure 11e).

**Figure 12:**
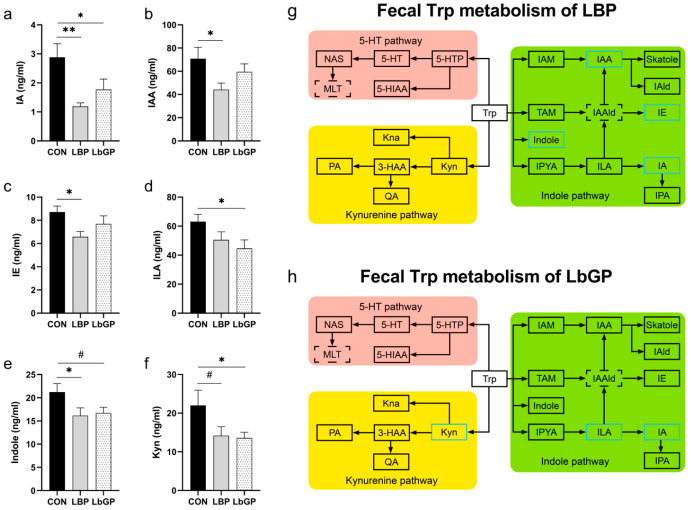
Effects of LBP and LbGP on Feces Metabolites of the Trp Metabolic Pathway on 42 D. (**a**) IA, indole acrylic acid; (**b**) IAA, indole-3-acetic acid; (**c**) IE, indole ethanol. (**d**) ILA, indole-3-lactic acid; (**e**) Indole, indole; (**f**) Kyn, kynurenine; (**g**) Fecal Trp metabolism of LBP; (**h**) Fecal Trp metabolism of LbGP. Black box: no statistical difference; blue box: decrease; dotted box: less than three samples were detected. . CON: control group (basal diet); LBP: basal diet supplemented with 20 mg/kg BW *Lycium barbarum* polysaccharides; LbGP: basal diet supplemented with 19 mg/kg BW *Lycium barbarum* glycopeptide. Data are presented as mean ± SEM. Asterisks (*) indicate statistically significant differences compared to baseline or control (* *p* < 0.05, ** *p* < 0.01), and the hash symbol (^#^) denotes a statistical trend (^#^ *p* < 0.10). For a complete list of abbreviations of all detected Trp-related metabolites, see Supplementary Materials Table S2.

As shown in Figure 12, analysis of specific metabolites in the Trp metabolic pathway in dog feces on 42 D indicated that both LBP and LbGP exerted regulatory effects on particular metabolites within this pathway. Specifically, in the LBP group, fecal indole acrylic acid levels were significantly lower than those in the CON group (*p* < 0.01, Figure 12a). Indole-3-acetic acid, indole ethanol, and indole levels were lower than those in the CON group (all *p* < 0.05, Figure 12b,c,e). Kyn levels showed a trend toward being lower than those in the CON group (*p* < 0.10, Figure 12f). In the LbGP group, indole acrylic acid, indole-3-lactic acid, and Kyn levels were significantly lower than those in the CON group (all *p* < 0.05, Figure 12a,d,f). Indole concentrations tended to be lower than those in the CON group (*p* < 0.10, Figure 12e).

## 4. Discussion

Recent studies have indicated that dietary supplementation with plant extracts serves as a non-invasive, relatively safe, and cost-effective nutritional intervention for alleviating cognitive deficits and anxiety-like behaviors in animals experiencing chronic stress [14,15,19,45,46,47]. Notably, LBP is a crude polysaccharide mixture, whereas LbGP is a purified glycopeptide. As demonstrated by Hou et al. (2024) in the context of *Alpinia zerumbet*, purification of a crude polysaccharide led to a homogeneous fraction with enhanced anti-inflammatory activity and distinct structural features, suggesting that purification can alter bioactivity through changes in molecular weight, monosaccharide composition, and glycosidic linkages [48]. With a high purity and a smaller molecular weight, LbGP (88 kDa) may exert stronger and mechanistically distinct regulatory effects on the host compared to LBP (10–2300 kDa) [17,21]. Therefore, we expected that LbGP might exhibit distinct or even enhanced biological activities compared to the parent LBP extract. Based on previously established dosages of LBP and LbGP [34,35], the present study evaluated the efficacy of LBP and LbGP in enhancing cognitive function and elucidating underlying mechanisms in laboratory-kenneled dogs.

### 4.1. Cognitive Ability Test

As the experimental dogs were laboratory poodles rather than socialized pets and had not received prior behavioral training, only six tasks from the original protocol were selected to evaluate cognitive performance [36]. Some dogs in the current study displayed low motivation for food rewards in novel environments, leaving five dogs per group that completed cognitive testing. Notably, all dogs excluded from the cognitive tests were females, which may be explained by the fact that females tend to be more anxious, cautious, and risk-averse than males, resulting in less social interaction in outdoor settings [49]. Despite the reduced sample size, significant treatment effects were still detected, demonstrating that both LBP and LbGP conferred cognitive benefits. While poodles are generally considered highly intelligent, the baseline cognitive performance in this study was lower than that reported in Labrador Retrievers [36], likely due to the housing environment [50,51]. Results of the current study indicated that both LBP and LbGP counteracted this impairment, improving the performance of poodles in tasks assessing inhibitory control, social cognition, and memory. Logical reasoning was only improved in LBP treated group, which is possibly mediated by the specific upregulation of serum BDNF by LBP [52,53,54]. BDNF is known to play a critical role in synaptic plasticity, learning, and higher-order cognitive functions, including reasoning and problem-solving [55]. Thus, the selective improvement in logical reasoning in the LBP group is consistent with its specific ability to elevate BDNF. Nevertheless, we cannot rule out the potential roles of other mechanisms (e.g., pathways involving increased levels of 5-HT and other neurotransmitters) [56], which warrant further investigation.

### 4.2. Gut Microbiota and SCFAs

In our study, both treatments influenced gut microbiota compared to control, but LBP exhibited a more pronounced effect on the beta diversity of the gut microbiota compared to LbGP. This difference may be attributed to the more homogeneous composition of LbGP, which is further purified and isolated from LBP, potentially resulting in a narrower spectrum of microbial modulation [20].

The present study found that LBP treatment significantly increased the relative abundance of *Faecalibacterium*, *Bacteroides*, and *Parasutterella*, while decreased the abundance of *Terrisporobacter* at genus level compared to the CON group. LEfSe analysis between CON and LBP groups identified *Romboutsia* and *Terrisporobacter* as biomarker taxa in the CON group, whereas *Faecalibacterium*, *Bacteroides*, *Acutalibacter*, and *Parasutterella* were enriched in the LBP group. Known for its anti-inflammatory properties, *Faecalibacterium* produces one of the SCFAs, butyrate to help maintain intestinal barrier integrity and mitigate inflammation [57]. Reduced abundance of *Faecalibacterium* has been associated with several disorders, including inflammatory bowel disease (IBD), colorectal cancer (CRC), dermatitis, and neurological conditions [58]. Moreover, certain strains of these bacteria have been demonstrated to influence the metabolites of the indole pathway, which is part of tryptophan metabolism, through the production of relevant enzymes (e.g., 2-amino-3-carboxymuconate semialdehyde decarboxylase) [59]. *Bacteroides* is a dominant genus in the gut microbiota that ferments complex carbohydrates to produce SCFAs such as acetate and propionate, which also exert anti-inflammatory effects [60]. Additionally, *Bacteroides* is a genus highly associated with tryptophan metabolism [61]. *Parasutterella* is implicated in purine metabolism and primary bile acid biosynthesis, and purine metabolites were shown to modulate immune responses and reinforce the gut mucosal barrier [62]. Previous study has reported a positive correlation between *Parasutterella* abundance and SCFA production [63]. In contrast, *Terrisporobacter* which is enriched in the CON group is considered a pathobiont associated with elevated serum lipids, dyslipidemia, oxidative stress, and reduced SCFA levels [64,65,66,67], indicating that LBP may help suppress potential pathogens in the gut. The other biomarker in the CON group, *Romboutsia*, is often considered a signature of gut health and abundant in healthy individuals [68,69]. Our results align with previous studies showing that LBP supplementation significantly reduces the relative abundance of *Romboutsia* in rats [70,71]. Interestingly, one study reported that a subspecies of *Romboutsia*, strain MY01, markedly increased the production of SCFAs during feed fermentation [72]. Thus, the reduction in *Romboutsia* induced by LBP may partially explain its fewer effects on fecal SCFA levels compared to LbGP.

In the LEfSe comparison between the CON and LbGP groups, no significant biomarkers were identified for the LbGP group, whereas *Escherichia-Shigella* and *Corynebacterium* were enriched in the CON group. *Escherichia-Shigella* is associated with numerous pathologies, including alcoholic cirrhosis, bacillary dysentery, and IgA nephropathy (IgAN) [73,74,75]. Its expansion in the gut may promote dysbiosis and inflammation, potentially triggering related diseases. Some studies have also linked *Escherichia-Shigella* to generalized anxiety disorder (GAD), with significantly higher abundance observed in GAD patients [76]. *Corynebacterium* is also another pathobiont that can cause diphtheria, skin infections, and respiratory tract infections [77,78,79]. Several studies have reported associations between *Corynebacterium* and Alzheimer Disease, depression, and anxiety-like behaviors [80,81]. Collectively, these findings support the potential mechanism of both LBP and LbGP in the amelioration of the cognitive impairment from chronic stress via the modulation of gut microbiota. The study by Li et al. (2025) found that specific bacteria, such as *Flavonifractor plautii* and its metabolites, are associated with certain metabolic diseases, supporting the view that specific compositional changes in the gut microbiota can directly influence host health status [82]. Therefore, the LBP- and LbGP-induced microbial shifts described above likely promote improvements in host metabolism and immune homeostasis, which in turn may play a role in the observed cognitive benefits.

As the fermentation products of gut microbiota, SCFAs have been shown to alleviate depressive symptoms with potential mechanisms of suppressing neuroinflammation and acting on receptors at the blood–brain barrier [83,84]. Several studies have reported that LBP supplementation can increase fecal SCFAs levels [85,86,87], yet the effect of LbGP in this regard remains largely unexplored. In the present study, compared to LBP, LbGP appeared to exert a more pronounced effect on enhancing fecal SCFAs levels. This suggests that LbGP may serve as a more efficient prebiotic that is readily utilized by gut microbiota for SCFAs production [88,89]. The effect of LbGP enhancing SCFAs observed in our study is consistent with several recent reports using dietary polysaccharides or prebiotics. For example, Ren et al. (2024) reported that the cognitive benefits of polysaccharide ORP-1 were accompanied by the reversal of memory- and synaptic plasticity-related protein levels and the inhibition of microglia-mediated synapse engulfment, highlighting the critical role of SCFAs in modulating neuroinflammatory processes [90]. Purification and fractionation of polysaccharides result in a more uniform molecular weight distribution and simplified monosaccharide composition, thereby significantly enhancing the fermentation efficiency of the gut microbiota [91]. Compared with high-molecular-weight polysaccharides, low-molecular-weight polysaccharides exhibit faster carbohydrate consumption rates and accelerate the production of short-chain fatty acids (SCFAs) [91]. Furthermore, polysaccharides with distinct structural features can be selectively utilized by specific gut microbiota through carbohydrate-active enzymes (CAZymes) [92]. This may explain why LBP, with its more complex composition, exerted a broader impact on the gut microbiota, whereas LbGP, a more homogeneous purified product, was directionally utilized by particular bacterial groups, resulting in more uniform fermentation products and a greater capacity in SCFA production. Studies have reported that in vitro fermentation of LBP increases the production of SCFAs, and experiments have shown that LBP exhibits a comparable capacity to inulin in producing SCFAs, with even better butyrate production [93]. However, research on the in vitro fermentation effects of LbGP remains limited, which represents one of the future research directions.

These results indicate that both LBP and LbGP can improve intestinal and overall health in dogs by modulating gut microbiota and increasing SCFA levels, although through probably distinct mechanisms. LBP appears to enrich beneficial bacteria, whereas LbGP may function more effectively in reducing harmful bacteria and enhancing microbial production efficiency of SCFAs.

### 4.3. Serum Biochemical Parameters

Throughout the trial, various serum biochemical parameters exhibited fluctuations within the normal reference range. No significant differences were observed between the experimental and CON groups, indicating that dietary supplementation with LBP or LbGP did not adversely affect the health of Poodles.

Within the LBP and LbGP groups, several parameters showed significant changes from baseline to post-treatment. For instance, reductions in ALB and TP levels were noted. The decline in TP may be attributed to the decrease in ALB, which was already near the upper limit of the normal range prior to intervention. Elevated ALB levels can be associated with conditions such as dehydration or chronic inflammation [94]. Supplementation with LBP and LbGP appeared to mitigate this elevation. In agreement, numerous studies have documented the anti-inflammatory properties of LBP and LbGP [95,96,97,98,99]. A previous study also reported that LBP ameliorated symptoms in a mouse model of Sjögren’s syndrome and rheumatoid arthritis [100,101].

Existing evidence indicates that chronic stress can significantly increase blood Ca concentrations in mice [102]. Hypercalcemia may lead to symptoms such as weakness, lethargy, polyuria, polydipsia, and anorexia in companion animals [103]. The reduction in serum Ca levels by the treatments observed herein also supports the stress mitigation and potentially other biological effects of LBP and LbGP. In addition, the observed reduction in AST levels is consistent with a 2023 study demonstrating that LBP ameliorated acute liver injury and the associated marked elevation in AST in dogs [30].

### 4.4. Oxidative Stress and Immune Function

Chronic stress leads to excessive production of reactive oxygen species (ROS), which can cause cell damage and various forms of cell death, such as apoptosis, autophagy, and ferroptosis, which has been closely associated with numerous neurological disorders [104]. Dai et al. (2023) have demonstrated that LbGP administration alleviated stress-induced anxiety by modulating oxidative stress and ferroptosis in the medial prefrontal cortex, probably through upregulating the expression of the *Sod* gene and restoring SOD activity [22,105]. Consistent with these findings, both LBP and LbGP supplementation increased serum SOD levels in our study, even though the increase was less pronounced in the LbGP group compared to the LBP group. Treatment of LbGP resulted in a significant elevation in serum GSH-Px activity, a key enzyme that reduces peroxides and inhibits lipid peroxidation in vivo [106]. Consistent with this, Ma et al. (2021) demonstrated that polysaccharide-induced elevation in SOD and GSH-Px activities effectively attenuated oxidative stress and preserved tissue integrity, providing direct evidence that enhanced antioxidant enzyme activity is a key mechanism underlying the protection against oxidative damage, a process intimately linked to cognitive function [107]. Therefore, both LBP and LbGP appear to alleviate oxidative stress in dogs by enhancing the activity of key peroxidase enzymes, with LbGP potentially offering a more comprehensive antioxidative profile due to its pronounced effect on GSH-Px. Serum CAT levels increased in all dogs over the experimental period, which may reflect a compensatory enhancement of antioxidant defense in response to the housing conditions. In agreement, liver CAT activity was shown to be significantly elevated in chronically stressed rats in the middle-to-late phases compared to the early phase [108]. Although these peroxidases may not provide a complete picture of the systemic antioxidant capacity, they offer valuable mechanistic insights into the potential pathways involved [109].

Compared to the CON group, both LBP and LbGP supplementation increased serum levels of IL-6 and IL-10 in dogs by the end of the trial. IL-6 appears to play complex, dual roles in cognitive ability. On one hand, it may exert neuroprotective effects by promoting the clearance of Alzheimer’s-associated plaques and tangles via astrocyte- and microglia-driven inflammatory responses [110]. On the other hand, IL-6 can activate the hypothalamic–pituitary–adrenal (HPA) axis, elevating cortisol levels that may promote neuroinflammation, hippocampal atrophy, and cognitive decline [111]. Given the positive effects of LBP and LbGP on cognitive performance observed in our study, it is plausible that the elevated IL-6 levels may exert beneficial neuromodulatory effects in dogs. One study showed that LBP promoted the maturation of dendritic cells (DCs) [112]. As a key cytokine secreted by mature DCs, IL-6 plays an essential role in promoting the differentiation of T follicular helper (Tfh) cells, which promote the humoral immune response by helping B cells differentiate into antibody-producing plasma cells and enabling the production of high-affinity antibodies [113]. Furthermore, studies using IL-6-deficient rats have also demonstrated the function of IL-6 in promoting nerve regeneration [114]. The increase in IL-6 observed in response to LBP and LbGP in our study may reflect a similar immunomodulatory mechanism. The anti-inflammatory cytokine IL-10 plays a critical role in preventing chronic inflammatory diseases [115,116]. The observed increase in IL-10 levels in our study is consistent with previous findings from multiple murine studies investigating LBP effects [117,118]. Although research on the effects of LbGP on IL-10 remains limited, the results from the present experiment suggest that LbGP likely influences IL-10 in a manner similar to LBP. At the end of the trial, serum levels of classic pro-inflammatory cytokines (i.e., TNF-α, IFN-γ, and IL-1β) in all dogs were significantly elevated compared to baseline. The experimental procedures and laboratory housing environment might be associated with the physiological changes in the dogs throughout the experimental period [119].

### 4.5. BDNF, Cortisol and NfL

BDNF is a crucial neurotrophin supporting synaptic plasticity, learning, and memory [55]. Numerous studies have indicated that low serum BDNF levels are associated with various neurological disorders, such as age-related cognitive impairment, Alzheimer Disease, and depression [120,121,122]. Crucially, chronic stress can suppress BDNF levels, impairing neurological function and increasing the risk of neurodegeneration [123]. In our study, dogs supplemented with LBP showed a significant increase in serum BDNF levels at Day 42 compared to Day 0. This result is consistent with several previous studies in other animal models demonstrating that LBP supplementation elevates hippocampal BDNF levels [19,124]. In contrast, LbGP supplementation did not significantly affect serum BDNF levels over time. To the best of our knowledge, no previous studies have explored the effect of LbGP on BDNF expression. This discrepancy between treatments suggests that LbGP may have a limited influence on BDNF, or it may require relatively longer intervention period than six weeks. Further investigations are required to clarify this observation.

As a critical hormone participating in the stress responses, sustained elevation in cortisol is often observed in individuals experiencing chronic stress, which can adversely affect dog health and behavior [125,126]. Salivary cortisol concentration correlates with plasma cortisol levels and reflects the response of the HPA axis, providing a reliable estimate of the available free cortisol [127]. Our results demonstrated that, in comparison to LBP, LbGP demonstrated a more pronounced effect on stress reduction, as evidenced by a significant decrease in salivary cortisol and a concurrent trend of reduction in serum cortisol. This suggests that LbGP is more effective in ameliorating chronic stress in dogs.

Neurofilament light chain (NfL) is a structural protein released upon axonal injury and its elevated plasma level has been observed in human neurological disorders such as Alzheimer Disease [128]. Consistently, serum NfL levels in dogs positively correlate with the severity of Canine Cognitive Dysfunction Syndrome (CDS), suggesting its potential as a biomarker for assessing cognitive function in dogs [129]. Other factors, such as age, body weight, and height were also shown to impact serum NfL concentrations in dogs [130]. In the present trial, serum NfL increased significantly over the experimental period, indicating that the experimental procedures and the housing environment might have induced negative effects on the neuroaxonal integrity in dogs. However, based on the test performance of dogs in the LBP and LbGP groups, serum NfL levels alone may not be regarded as a sole indicator of cognitive ability. NfL can serve as a biomarker of axonal injury but cannot predict cognitive function on its own. The neurotrophic cascades dependent on BDNF or nerve growth factor (NGF), are of particular importance for maintaining the integrity of synaptic plasticity [131]. Synaptic plasticity constitutes the neural basis of adaptive behavior [132], which may explain why LBP, compared to LbGP, improves the accuracy in the logical reasoning test.

### 4.6. Targeted Metabolome

Based on previous studies which indicate a close relationship between Trp metabolism and cognitive function, we hypothesized that Trp metabolism might be a key target through which LBP and LbGP alleviate cognitive decline in dogs [133]. In the present study, LBP supplementation upregulated the concentration of serum indole while reducing fecal indole-3-acetic acid, indole, indole ethanol, and indole acrylic acid compared to the CON group. It has been suggested that LBP may promote the production of indole derivatives through the regulation of gut microbiota and the subsequent absorptive process by the host [134]. Multiple indole compounds can bind to the aryl hydrocarbon receptor (AhR), which is expressed in various host cell types, especially including those epithelial and immune cells in the intestinal mucosa. By regulating the renewal of intestinal epithelium, barrier function, and the activity of various intestinal immune cells, AhR signaling pathway contributes to the maintenance of intestinal mucosal homeostasis [134]. Therefore, we speculate that LBP may predominantly act through the indole pathway to modulate inflammation, antioxidant capacity, and immune function, thereby contributing to the amelioration of cognitive impairment in dogs [135].

In contrast, LbGP supplementation upregulated serum concentrations of Trp, 5-HT, and indole-3-lactic acid, downregulated serum indole ethanol levels, and decreased fecal Kyn, indole-3-lactic acid, and indole acrylic acid concentrations compared to the CON group. These results suggest that LbGP may primarily influence the 5-HT pathway of Trp metabolism. Stress is known to overactivate the Kyn pathway, leading to the accumulation of neurotoxic metabolites such as quinolinic acid (QA) and the excessive depletion of peripheral Trp [136,137,138]. Although no significant changes in serum metabolites of Kyn pathway were detected in the LbGP group in this study, both LBP and LbGP reduced fecal Kyn levels, with a more pronounced effect observed in the LbGP group. Fecal Kyn originates from host synthesis and excretion, microbial production, and dietary sources, therefore reduced fecal Kyn levels may reflect the downregulation of the Kyn pathway [139,140]. Interestingly, a previous study found that a significant increase in intestinal indole derivatives activated colonic AhR, promoted the Kyn pathway, and inhibited the 5-HT pathway, which may explain why LBP did not significantly modulate the 5-HT pathway as observed with LbGP [141]. As discussed earlier, polysaccharides with distinct structural features can be selectively utilized by specific gut microbiota [92]. The more homogeneous composition of LbGP may be preferentially fermented by certain bacterial populations, leading to increased Trp availability and subsequent upregulation of host 5-HT metabolism. In contrast, the more complex composition of LBP likely favors the production of indole pathway metabolites. Together, these findings imply that Trp metabolism may be the key pathway involved in the effects of LBP and LbGP on cognitive function, potentially via the MGB axis. However, further studies are required to elucidate the specific mechanisms.

### 4.7. Limitations and Future Directions

The specific subjects chosen in the current study, with certain breed (Poodles), age range, and life experience may limit the generalizability of the results. In addition, we acknowledge that the sample size per group was relatively small (n = 6, with 3 males and 3 females), which limited the statistical power for sex-stratified analyses. The small sample size increases the risk of random variation and limits the generalizability of the findings. Moreover, the use of the least significant difference (LSD) post hoc test for multiple comparisons prioritizes sensitivity, which may lead to a slightly increased chance of detecting differences that might not be confirmed in larger or more conservative analyses. Accordingly, our findings should be interpreted with appropriate caution. Future research should aim to validate these findings across different dog populations, in longer-term trials, and with dose–response designs, which may better elucidate the precise mechanisms of action of LBP and LbGP on dog cognitive function.

## 5. Conclusions

This study demonstrates that dietary supplementation with LBP or LbGP improves cognitive performance in laboratory-kenneled poodles, potentially through distinct mechanisms involving immunomodulation, antioxidant enhancement, gut microbiota remodeling, and differential regulation of tryptophan metabolism (indole versus 5-HT pathways). These findings suggest that both LBP and LbGP hold promise as functional feed additives to support canine cognitive health. However, the small sample size, single breed, and lack of long-term follow-up limits the generalizability of the results. Future studies in broader dog populations with extended trial durations and dose–response designs are warranted to validate and extend these observations.

## Figures and Tables

**Table 1:** Effects of LBP and LbGP on serum biochemical parameters in dogs.

Item	Range	Days	Groups			*p*-Value		
			CON	LBP	LbGP	Time	TRT	Time × TRT
ALB (g/L)	23.0–40.0	0	33.63 ± 3.29	35.75 ± 5.15 **	37.08 ± 2.16 *	0.003	0.459	0.029
		42	33.95 ± 0.67	32.75 ± 3.99 **	34.80 ± 1.98 *			
TP (g/L)	49.0–82.0	0	72.13 ± 4.38	76.43 ± 6.34 **	70.07 ± 3.63	0.032	0.35	0.027
		42	72.68 ± 5.02	68.77 ± 4.86 **	68.93 ± 3.37			
GLB (g/L)	19.0–45.0	0	38.83 ± 5.10	40.67 ± 8.19	32.97 ± 4.58	0.371	0.124	0.147
		42	39.08 ± 5.00	36.05 ± 5.91	34.13 ± 2.67			
A/G	–	0	0.87 ± 0.17	0.92 ± 0.27	1.15 ± 0.20	0.297	0.143	0.186
		42	0.87 ± 0.14	0.94 ± 0.24	1.03 ± 0.10			
AST (U/L)	0–50	0	26.67 ± 10.37	29.67 ± 8.16 *	29.33 ± 11.09	0.09	0.861	0.050
		42	29.83 ± 10.87	21.17 ± 5.49 *	25.00 ± 11.31			
ALT (U/L)	5–125	0	52.00 ± 19.15	53.17 ± 17.93	46.17 ± 19.86	0.539	0.479	0.955
		42	48.00 ± 9.76	51.50 ± 29.54	39.67 ± 15.63			
AMY (U/L)	400–1500	0	692.50 ± 72.38	711.17 ± 64.97	727.50 ± 142.82	0.05	0.858	0.214
		42	680.67 ± 138.14	677.83 ± 100.55	601.83 ± 125.15			
CK (U/L)	10–200	0	117.83 ± 45.79	174.17 ± 49.28	136.83 ± 39.49	0.44	0.25	0.733
		42	95.17 ± 30.53	132.17 ± 40.29	119.00 ± 26.86			
CREA (μmol/L)	28.0–159.0	0	72.00 ± 8.69	77.90 ± 8.32	81.17 ± 19.67	0.47	0.538	0.222
		42	76.72 ± 9.13	69.58 ± 4.41	78.27 ± 14.12			
BUN (mmol/L)	2.50–9.60	0	5.20 ± 1.54	4.63 ± 0.43	5.13 ± 1.18	0.634	0.360	0.803
		42	5.69 ± 1.7	4.86 ± 0.42	4.98 ± 1.14			
BUN/CR	16.000–218.000	0	72.18 ± 19.51	59.71 ± 5	64.51 ± 13.18	0.174	0.145	0.217
		42	73.37 ± 16.36	69.94 ± 6.67	64.04 ± 12.55			
GLU (mmol/L)	4.11–7.94	0	4.48 ± 0.98	4.40 ± 1.13	4.41 ± 0.53	0.467	0.877	0.513
		42	4.21 ± 0.53	4.56 ± 0.78	4.13 ± 0.77			
TG (mmol/L)	0.00–1.13	0	0.75 ± 0.23	0.79 ± 0.24	1.00 ± 0.54	0.528	0.192	0.991
		42	0.81 ± 0.18	0.88 ± 0.25	1.08 ± 0.52			
Ca (mmol/L)	1.98–3.00	0	2.29 ± 0.21	2.46 ± 0.19 *	2.48 ± 0.12 *	0.013	0.258	0.099
		42	2.30 ± 0.07	2.33 ± 0.08 *	2.32 ± 0.12 *			
P (mmol/L)	0.81–2.19	0	1.06 ± 0.26	1.15 ± 0.34	1.14 ± 0.33	0.003	0.813	0.453
		42	1.39 ± 0.14	1.40 ± 0.12	1.27 ± 0.19			
Ca × P (mmol/L)	–	0	2.43 ± 0.71	2.84 ± 0.88	2.84 ± 0.85	0.027	0.711	0.338
		42	3.19 ± 0.26	3.27 ± 0.34	2.94 ± 0.53			
AST/ALT	–	0	0.51 ± 0.11 ^#^	0.61 ± 0.25 *	0.68 ± 0.27	0.554	0.559	0.02
		42	0.61 ± 0.18 ^#^	0.47 ± 0.20 *	0.66 ± 0.28			

**Note:** 0 D refers to the start of the experiment (Day 0), and 42 D indicates the end of the experimental period (Day 42). CON: control group (basal diet); LBP: basal diet supplemented with 20 mg/kg BW *Lycium barbarum* polysaccharides; LbGP: basal diet supplemented with 19 mg/kg BW *Lycium barbarum* glycopeptide. ALB, albumin; TP, total protein; GLB, globulin; A/G, albumin-to-globulin ratio; AST, aspartate aminotransferase; ALT, alanine aminotransferase; AMY, amylase; CK, creatine kinase; CREA, creatinine; BUN, blood urea nitrogen; BUN/CR, BUN-to-creatinine ratio; GLU, glucose; TG, triglycerides; Ca, calcium; P, inorganic phosphorus; Ca × P, calcium-phosphorus product; AST/ALT, AST-to-ALT ratio. Data are presented as mean ± SD. Asterisk (*) denotes a significant difference within the same group over time (* *p* < 0.05, ** *p* < 0.01), and the hash symbol (^#^) denotes a statistical trend within the same group over time (^#^ *p* < 0.10).

## Data Availability

The 16S rRNA data were deposited in the NC-BI repository, accession number: https://www.ncbi.nlm.nih.gov/, accessed on 5 December 2025, PRJNA1375475.

## Supplementary Materials

The following supporting information can be downloaded at: https://www.mdpi.com/article/10.3390/microorganisms14040940/s1, Table S1: The ingredients and analyzed chemical composition of basal diet. Table S2. Standard compounds of metabolites in tryptophan metabolism pathway.

## Abbreviations

The following abbreviations are used in this manuscript:

5-HT	5-hydroxytryptamine
A/G	albumin-to-globulin ratio
ALB	albumin
ALT	alanine aminotransferase
AMY	amylase
AST	aspartate aminotransferase
BCFAs	branched-chain fatty acids
BDNF	brain-derived neurotrophic factor
BUN	blood urea nitrogen
BW	body weight
Ca	calcium
CAT	catalase
CK	creatine kinase
CON	control group
CREA	creatinine
GLB	globulin
GLU	glucose
GSH-Px	glutathione peroxidase
HPA	hypothalamic–pituitary–adrenal
Kyn	kynurenine
IFN-γ	interferon-gamma
IL-1β	interleukin-1 beta
IL-6	interleukin-6
IL-10	interleukin-10
LBP	*Lycium barbarum* polysaccharides
LbGP	*Lycium barbarum* glycopeptide
LDA	linear discriminant analysis
LEfSe	linear discriminant analysis Effect Size
NfL	neurofilament light chain
OTU	operational taxonomic unit
P	inorganic phosphorus
PCoA	principal coordinates analysis
SCFAs	short-chain fatty acids
SD	standard deviation
SEM	standard error of the mean
SOD	superoxide dismutase
TG	triglycerides
Tfh	T follicular helper cells
TNF-α	tumor necrosis factor-alpha
TP	total protein
TRT	treatment administrations
