# Supplementary material for: Cognitive Improvement and Microbiota–Gut–Brain Axis Regulation by Lycium barbarum Polysaccharides and Glycopeptide in Laboratory-Kenneled Poodles
Source: Microorganisms. 2026 Apr 21;14(4):940. doi: 10.3390/microorganisms14040940 (PMC13118899; doi:10.3390/microorganisms14040940)
Supplement: Supplementary file 1 [file microorganisms-14-00940-s001.zip › microorganisms-4239997-supplementary.pdf]

Table S1 The ingredients and analyzed chemical composition of basal diet

Items	Inclusion (%)
Ingredients (as-is basis)	
Chicken meal	32.00
Duck meal	20.00
Sweet potato meal	12.00
Tapioca meal	12.00
Brewer's yeast meal	5.00
Alfalfa grass granules	5.00
Chicken oil	3.00
Fish oil	2.00
Flaxseed	1.00
Pumpkin meal	1.00
Apple meal	0.50
Analyzed composition	
DM (%)	96.31
CP (% DM)	37.27
EE (% DM)	18.12
CF (% DM)	2.61

Premix: lysine, DL-methionine, fructooligosaccharides, vitamin A, vitamin D3, vitamin E, vitamin B1, vitamin B2, vitamin B6, vitamin B12, D-biotin, D-pantothenic acid, folic acid, niacin, ferrous sulfate, copper sulfate, manganese sulfate, zinc sulfate, calcium iodate, sodium selenite, and calcium hydrogen phosphate. DM, dry matter; CP, crude protein; EE, ether extract; CF, crude fiber.

Table S2. Standard compounds of metabolites in tryptophan metabolism pathway.

Abbreviation	Compound Name	HMDB
3-HAA	3-Hydroxyanthranilic acid	HMDB0001476
5-HIAA	5-Hydroxyindoleacetic acid	HMDB0000763
5-HT	5-Hydroxytryptamine	HMDB0000259
5-HTP	5-Hydroxytryptophan	HMDB0000472
IA	Indole acrylic acid	HMDB0000734
IAA	Indole-3-acetic acid	HMDB0000197
IAAld	Indole-3-acetaldehyde	HMDB0001190
IAld	Indole-3-aldehyde	HMDB0029737
IAM	Indole-3-acetamide	HMDB0029739
IE	Indole ethanol	HMDB0003447
ILA	Indole-3-lactic acid	HMDB0000671
Indole	Indole	HMDB0000738
IPA	3-Indolepropionic acid	HMDB0002302
IPYA	Indole-3-pyruvate	HMDB0600067
Kna	Kynurenic acid	HMDB0000715
Kyn	Kynurenine	HMDB0000684
MLT	Melatonin	HMDB0001389
NAS	N-Acetyl-5-hydroxytryptamine	HMDB0001238
PA	Picolinic acid	HMDB0002243
QA	Quinolinic acid	HMDB0000232
Skatole	Skatole	HMDB0000466
TAM	Tryptamine	HMDB0000303
Trp	Tryptophan	HMDB0000929
